# Origin of “Conscientious Objection” in Health Care: How Care Denials Became Enshrined into Law Because of Abortion

**DOI:** 10.1017/jme.2025.46

**Published:** 2025

**Authors:** Christian Fiala, Joyce Arthur, Amelia Martzke

**Affiliations:** 1 Department of Women’s and Children’s Health, Karolinska Institutet, Stockholm, SE, Sweden; 2 Independent Scholar, Vancouver, CA, Canada; 3 University of British Columbia, Faculty of Medicine (MS1), Kelowna, BC, Canada

**Keywords:** abortion, conscientious objection, reproductive healthcare, belief-based care denial, conscientious objectors

## Abstract

The United Kingdom was the first country to legalize the refusal to provide health care in the name of “conscientious objection”, allowing doctors to refuse to provide abortions based on personal or religious beliefs.

A historical review into the origins and motivation behind the “conscientious objection” clause in the 1967 Abortion Act found that Parliamentarians and the medical profession wanted to preserve doctors’ authority over patients, protect objecting doctors from liability, and appease religious anti-abortion beliefs.

These factors point to an unprincipled basis for the introduction of “conscientious objection” into healthcare, which ultimately came at the expense of patients’ rights and health. The “conscience clause” also represented a negation of basic ethical directives in medical practice including patient autonomy and physicians’ fiduciary duty to patients. The term “conscientious objection”— borrowed from the military but misapplied to healthcare — helped mask the practice as a moral “right” of doctors, even while it disregarded patients’ health and dignity.

Refusing to provide treatment on the basis of “conscience” is harmful and discriminatory, and should be phased out gradually using disincentives and other measures to encourage objectors to choose other fields.

## Introduction

How did the practice of denying care under “conscientious objection” in health care arise, and what were the motivations for the application of this practice to abortion care in particular?

Along with some other scholars and researchers, we subscribe to the “incompatibility thesis,” which holds that medical professionalism and the practice of “conscientious objection” are incompatible.^1^ We argue against the practice based on evidence that it violates human rights and medical ethics, constitutes a misuse of physician authority, undermines gender equality, and causes harms to patients. It should not be considered a “right” and should be phased out in a gradual process (which does not involve forcing doctors to provide abortion).

Understanding how “conscientious objection” became a part of medical practice in the first place may help shed light on its contradictions and motivate policymakers and legislators to initiate change. When exploring the history of the term “conscientious objection” as used in health care, we found that the world’s first modern “conscientious objection” law was passed in the United Kingdom in 1967, allowing doctors to refuse to provide abortion care upon its legalization.

We discuss how that term was mistakenly borrowed from military conscientious objection despite the lack of similarity with healthcare refusals, then draw connections between the ethical problems with the initial adoption of “conscientious objection” in healthcare by the United Kingdom, and the resulting harms of the practice across the world.

### Defining terms

The term “conscientious objection”^2^ in health care can be defined as the refusal by a health care professional to provide a legal, patient-requested medical service or treatment that falls within the scope and qualifications for their field, based on their personal or religious beliefs.^3^ Alternative terms suggested by those who oppose the practice include refusal to treat, denial of care, and “dishonorable disobedience.”^4^ This paper will generally use the term “belief-based care denial,”^5^ whether rooted in religious faith or other personal beliefs.

### Reproductive health care context

Refusing to provide care due to one’s personal beliefs is uncommon outside of reproductive health care. It was first specifically legalized in the case of abortion and is still used mostly against pregnant people with an unwanted pregnancy, indicating a selective discrimination against women^6^ and gender diverse people. Care denials also occur for contraception,^7^ sterilization,^8^ and fertility treatments,^9^ as well as medical assistance in dying and organ donation.^10^ However, medical assistance in dying remains illegal in most countries^11^ and the frequency of organ donation is low^12^ compared to reproductive care.

Belief-based denials of abortion care are often expanded to institutions, regions, or whole countries, even though only individuals have a conscience. In Italy,^13^ Austria,^14^ South Africa,^15^ Croatia,^16^ and many countries in Latin America,^17^ a significant proportion of doctors and hospitals — too often a majority — claim the “right” of conscience to refuse to do abortions.

### Early laws and policies governing belief-based care denials

The United Kingdom was the first country in the world to officially allow care denials based on “conscientious objection” by health care professionals, in the 1967 Abortion Act that legalized abortion.^18^ Six years later, in 1973, the United States passed the Church Amendments granting doctors and private hospitals the option to deny care due to personal or religious beliefs.^19^ This occurred just months after the US Supreme Court’s Roe v. Wade decision that legalized abortion nationwide.

Scholar Mark Wicclair notes that he was unable to find any academic articles on “conscientious objection” in health care prior to the 1960s.^20^ This indicates that the concept of legally exempting health care professionals from their duty of care because of their personal beliefs was a novel one, implemented in response to just one newly-legalized treatment — abortion.

Before the 1960s, it appears that unregulated care denials were the norm because historically, “doctors made the decisions; patients did what they were told.”^21^ A prevailing system of medical paternalism meant that doctors could deny care without consequences. However, in the aftermath of World War II, human rights and the value of individual autonomy gained ascendancy, and medicine became more patient-directed. Perceptions of medical authority and professionalism changed,^22^ which may have led to resistance by doctors who wanted to retain the ability to deny care. Perhaps this became more pressing with the legalization of abortion — a practice that was opposed by many. In this sense, the official institutionalization of belief-based care denial can be seen as a partial recapturing of doctors’ authority over patients and a rejection of patients’ rising claims to self-determination in democratic societies.

Today, about eighty-seven jurisdictions around the world have policies or laws that allow belief-based denial of abortion care.^23^ Most countries that do *not* regulate the practice have strict abortion laws or bans, which means that the laws themselves act as a form of institutional belief-based care denial — by the government instead of healthcare facilities.

## Military Conscientious Objection

Conscientious objection (CO) originated in the military and can be defined as “the position taken by individuals who oppose participation in war on the basis of their religious, moral, or ethical beliefs.”^24^ One of the first known uses of the term “conscientious objection” dates to 1841, in a New York government report on the militia.^25^

Although pacifism itself has a much longer history, including by Quakers and Mennonites during the 18^th^ century,^26^ the modern form of military CO did not become common until the First World War when it was legalized in 1917 in the US with the Selective Service Act, after campaigns by objectors.^27^ The Act defined a “conscientious objector” as someone with “a firm, fixed, and sincere objection to participation in war in any form or the bearing of arms, by reason of religious training and/or belief.” However, protections were limited to members of well-established religious groups with doctrines against participation in combat, such as Quakers.^28^

A comparison of “conscientious objection” in health care and CO in the military reveals fundamental differences.^29^ Soldiers are drafted into compulsory service in a subordinate position, while health care professionals who deny care based on personal beliefs enjoy a position of power and authority. Soldiers must justify their conscientious stance before a tribunal and accept punishment or alternate service in exchange for exercising their CO. In contrast, health care professionals usually face no obligation to justify their refusals, rarely face consequences for denying care, and often retain their positions and salaries,^30^ as well as escape stigma or harassment by avoiding abortion provision. Soldiers are conscripted to kill others for the economic or political benefit of those in power, while doctors have a fiduciary duty to patients and are obligated to preserve their health and lives — indeed, they exercise a monopoly on health care and are in a position of power, since patients are generally unable to access necessary care outside the medical system and cannot perform the intervention themselves.

Finally, killing a living person in war should not be equated with halting the development of a gestational sac or fetus, which is still a dependent and integral part of the pregnant person’s body and not an individual human being despite antiabortion views to the contrary. Belief-based denials can put patients’ lives and health at risk for the sake of a fetus or embryo, which is explicitly given the same legal status as a person in the constitutions of only a handful of countries, such as El Salvador^31^ and Honduras.^32^

Given these major differences, how did the term “conscientious objection” come to be adopted in health care?

## Conscientious Objection in the UK’s 1898 Vaccination Act

In the context of health care, the term “conscientious objector” first appeared in the United Kingdom’s 1898 Vaccination Act.^33^ The original 1853 version of the Act made vaccination obligatory for all infants, and subsequently all children up to age 14, invoking fines and penalties against any parents or guardians who refused to have their children vaccinated.^34^ Opposition to this Act was strong, resulting in an 1898 amendment that allowed for CO to vaccination. This amendment enabled parents or guardians who did not believe vaccination was effective or safe to acquire a certificate of their conscientious objection in order to be exempt from fines or penalties.^35^

Notably, the CO clause of the 1898 Vaccination Act applied only to parents and guardians, not medical practitioners — in other words, physicians could not refuse to perform vaccinations. Therefore, the Vaccination Act’s CO clause is similar to military CO as both were conditional exemptions from a compulsory government requirement imposed on the general citizenry. In contrast, “CO” in reproductive health is carried out by privileged professionals who voluntarily entered their occupation.

## United Kingdom’s 1967 Abortion Act

The first time that medical practitioners were legally allowed to refuse to provide care based on religious beliefs was in relation to abortion. Section 4 of the UK’s 1967 Abortion Act^36^ is entitled “Conscientious objection to participation in treatment.” It reads:

(1) Subject to subsection (2) of this section, no person shall be under any duty, whether by contract or by any statutory or other legal requirement, to participate in any treatment authorised by this Act to which he has a conscientious objection: Provided that in any legal proceedings the burden of proof of conscientious objection shall rest on the person claiming to rely on it.

(2) Nothing in subsection (1) of this section shall affect any duty to participate in treatment which is necessary to save the life or to prevent grave permanent injury to the physical or mental health of a pregnant woman.

(3) In any proceedings before a court in Scotland, a statement on oath by any person to the effect that he has a conscientious objection to participating in any treatment authorised by this Act shall be sufficient evidence for the purpose of discharging the burden of proof imposed upon him by subsection (1) of this section.

### Early drafts of the UK abortion bill

The private member bill that led to the 1967 Abortion Act was introduced by then-Liberal MP David Steel. However, this was not the first attempt to legalize abortion in the UK. While no government-sponsored bill was ever put forward, six private member bills preceded Steel’s bill, with a variety of grounds for the termination of pregnancy. The first bill was introduced in 1952 by Joseph Reeves, followed by Lord Amulree in 1954, Kenneth Robinson in 1961, Renée Short in 1965, Lord Silkin in 1965 and 1966, and Simon Wingfield Digby in 1966.^37^ These bills failed to pass, not simply due to lack of support but because the MPs who proposed them did not have favorable ballot slots in Parliament for private member bills, and very little time was available for debate.^38^ Further, these bills were “defeated largely by the delaying tactics of hostile Roman Catholic MPs,”^39^ who purposely exhausted the allocated debate time.^40^

### Glanville William’s early draft bill

“Conscientious objection” was first mentioned in an even earlier draft of an abortion bill by Glanville Williams, published in Alice Jenkin’s 1960 book *Law for the Rich.* ^41^ Williams was a Cambridge legal scholar and president of the Abortion Law Reform Association.^42^ He drafted many of the UK’s abortion bills, including those introduced by Joseph Reeves, Lord Amulree, Kenneth Robinson, Renée Short, and Lord Silkin.^43^

Williams’ draft bill (circa 1960 or earlier), which was never introduced in Parliament, states:

“A registered medical practitioner shall not be deemed to be guilty of a breach of duty if, because of a conscientious objection, he fails to advise or perform a termination of pregnancy declared to be lawful by this Act, provided that he believes in good faith that his conscientious objection is known to the patient whom he is attending or to the person whose consent to the operation is required by law on her behalf.”^44^

Williams specified that this clause was intended to protect any individuals with so-called “conscientious objections” to abortion, specifically Roman Catholic practitioners.^45^

### David Steel’s abortion bill

Of all the abortion bills, David Steel’s was the first to include an explicit clause allowing doctors to deny care due to “conscientious objection.” The clause was added to the bill later as an amendment.

Such “conscience” protections had also been acknowledged by Lord Silkin with his first abortion bill. Although his bill did not contain a conscience clause, he declared in a parliamentary debate in 1965 that “neither the medical profession nor the patients are going to be compelled against their conscience to accept abortion”. In this same debate, Silkin proclaimed that “… the Bill does not compel any medical practitioner to carry out an abortion. If he has a conscientious objection to doing so, he is under no obligation at all to carry out an abortion at the request of a patient.”^46^

### Rationale for adopting term “conscientious objection”

Hence, the concept of refusing to provide abortions based on personal or religious beliefs existed well before Steel’s 1967 Abortion Act, and the term “conscientious objection” was adopted for this purpose from at least the early 1960s. Why did this occur, given the dissimilarity with CO as allowed in the military and in the UK’s 1898 Vaccination Act? The most likely inference is that it came from Glanville Williams himself because he was a conscientious objector during World War II^47^ and used the phrase in his early draft of an abortion bill.

Otherwise, no explicit rationale for adopting the term “conscientious objection” in the Abortion Act was ever stated in the Parliamentary record.^48^ In the transcripts of the debates on Steel’s bill, three MPs did make comparisons to conscientious objection for military service in the context of military tribunals that tried objectors, and opined on whether health care workers should also be required to justify their beliefs.^49^

## Parliamentary Debates on the “CO” clause

We conducted a targeted review of selected sources that were most likely to yield relevant information on how the “CO” clause became part of the 1967 Abortion Act. Issues of the *British Medical Journal* (BMJ) from April 1966 to April 1968 were reviewed, and 20 articles or letters were found that made reference to the issue of “conscientious objection” or related terms in the context of legalizing abortion.^50^ Parliamentary Hansard debates from July 1966 to October 1967 were reviewed, seven of which contained discussion pertaining to “CO” in relation to the proposed Abortion Act.^51^ A search of other publications on the issue yielded over a dozen books and articles that discussed the origin of the United Kingdom’s “CO” clause.

Over the course of the bill’s passage, Parliamentarians held lengthy debates on several main topics: the definition of conscience and whether it should be broader than simply religious conscience, whether objectors should need to prove their objection and how, and whether doctors could be punished or prosecuted for not providing abortions. The answers were ultimately “Yes,” “No,” and “No,” respectively,^52^ although the last answer was conditional based on the final clause’s requirement that objectors must “save the life or prevent grave permanent injury to the physical or mental health of a pregnant woman.”

In both the BMJ articles and the Hansard debates, a significant number of comments and concerns were expressed regarding religion, with much deference given to Catholic religious beliefs against abortion. The House of Lords included many bishops and clerics, and many MPs were themselves deeply religious. Some feared that the UK law would obligate doctors to perform abortions, which were considered morally questionable and against Catholic religious doctrine — in the words of MP Peter Mahon: “the killing of an unborn child.”^53^ In the Hansard debate from July 13, 1967, MP Bernard Braine said: “There are thousands of Roman Catholic doctors who may well have a clear conscientious objection on religious grounds and who may feel themselves in hazard of litigation if they do not agree to terminations of pregnancy.”^54^ A letter to the BMJ by Dr. Myre Sim stated: “This clause was intended to protect those doctors (and nurses) with strong religious objections to abortion” although he also declared: “… a medical conscience should not require a special dispensation but is our main *raison d’être* as a profession.”^55^

Opposition to the “CO” clause was expressed by a small minority of voices in the BMJ and by Parliamentarians. Among them, an April 16, 1966 letter to the BMJ questioned a gynecologist’s right to deny care on the “grounds of moral prejudice” saying “It is clearly wrong for a person not prepared to perform abortions to follow a profession which requires him to do so.”^56^ Another writer said: “… the interest of the patient comes first and … the personal views of the doctor on any issue which is not purely medical should never be allowed to interfere with his judgment of fact.”^57^ MP Kenneth Robinson said in the July 13, 1967 Parliamentary debate: “I do not think it is universally accepted that a conscience Clause is necessary in the Bill. I consider that it is unnecessary, and I rather think that it is undesirable. This view is shared by some leaders of the medical profession.”^58^

Since the conscience clause was an amendment to Steel’s initial bill, several changes to the amendment were proposed during Parliamentary debates.^59^ One called for deleting the requirement that “in any legal proceedings the burden of proof of conscientious objection shall rest on the person claiming to rely on it.” MPs expressed concerns that this represented an inappropriate shifting of the burden of proof to the defendant instead of the prosecution, that it was unclear what kind of proof would be sufficient, and that it might disadvantage non-religious people as well as those unable to articulate their personal beliefs.^60^ The suggested amendment was rejected on the basis that military objectors also have to justify their stance and their reasons are not always based on religious beliefs.^61^ Another amendment proposed that “no person [shall be] deprived of, or be disqualified from, any promotion or other advantages by reason of the fact that he has such conscientious objection.”^62^ It was rejected on the basis that hospitals should be given discretion when it comes to staff duties to provide health care, and that “discrimination” against objectors would not be widespread in any case.^63^

## Why Was the “CO” Clause Included in UK’s Abortion Act?

The “conscientious objection” clause was incorporated into the 1967 *Abortion Act* for several reasons, with professional, political, and Catholic groups influencing its inclusion.

### Protection of health care workers

According to the Hansard transcripts, one key aim of the clause was to protect objecting medical practitioners from being “forced” to provide abortions and any criminal and civil liability if they refused.^64^

The British Medical Association (BMA) and other groups saw the “CO” clause as a critical measure to protect doctors’ decisional authority over patients.^65^ The idea that a woman could make an independent decision to terminate an unwanted pregnancy seemed intolerable to many physicians and medical associations. For example, the BMA in its 1966 report on Steel’s abortion bill insisted that “the ultimate decision on whether to advise termination rests with the doctors in charge of the case….”^66^ According to Gleeson:^67^

“Despite its success, however, and despite its trenchant sustained offensive, the ALRA [Abortion Law Reform Association] lost control of the Abortion Act as the BMA assumed moral and scientific authority throughout the course of the campaign for reform. The ALRA was inspired to legitimise its own position by way of medical authority, but its promotion of medical hegemony came to secure the full medicalisation of the Bill, contra much of the ALRA agenda.”

MP Bernard Braine and Lord Amulree noted that the “conscientious objection” clause was included at the request of the Royal College of Nurses, the Royal College of Midwives, the Association of Hospital Matrons, and the General Nursing Council in order to defend the position of medical professionals who may be asked to participate in terminations against their consciences.^68^ However, the MP who introduced the bill, David Steel, disputes that it was added because of these groups’ requests (see below).

Some assert that the “CO” clause was included as a pragmatic compromise with conservative and religious MPs to ensure that the Abortion Act was passed,^69^ although David Steel disputes this as well (more below). The claim is that the clause facilitated the approval of the Act because the supporters of legal abortion could obtain the backing of some opposing physicians and politicians by reassuring them that doctors would not have to be personally involved and would be protected from potential liability if they refused to provide abortion care.^70^ It does appear that pro-choice campaigners accepted the clause as the price to be paid for securing support for abortion law reform from conservative politicians and doctors.^71^

### Catholic and religious influence

Catholic religious demands were the primary impetus for adding the “CO” clause, which later came to be supported by MPs and medical groups for additional reasons. According to David Steel, the clause was written into the Act following multiple discussions he had with priests at St. Andrew’s College, a Catholic seminary in his constituency of Drygrange Scotland. In a 2018 documentary interview,^72^ as well as personal correspondence by email, Steel said:

“… during the passage of the bill, I had many conversations, at least three, with a Catholic seminary in my constituency. They were creedal priests, and they of course were very strongly opposed to the bill that I was introducing. So after these three discussions, I agreed that we should have a conscience clause, so that no Catholic or anybody who had a rooted objection to abortion would ever be required to take part in the operation. And that was the origin of it. So when the bill was going through the House of Commons, we passed an amendment to add the conscience clause into the bill. And everybody welcomed that.”^73^

“I realised that the law ought to respect the rights of those who (mistakenly but fervently!) equate abortion with murder. They [the seminary priests] were pleased at the outcome ….” ^74^

“It is not true that the CO clause was introduced to get the bill passed, though it possibly did influence some in the medical bodies …”^75^. “… these bodies embraced the idea of a conscience clause after the concept became public.”^76^

Steel had previously shared his views on the origin of the “CO” clause in a 2017 op-ed for The Independent:^77^

“There is always a problem with those who believe that human life begins at the moment of conception rather than the moment of birth. That remains the official doctrine of the Roman Catholic Church. It was to respect their view that during the passage of the Act I introduced the conscience clause permitting doctors and nurses to avoid participating in abortions, but of course that small minority who cling passionately to that view are not obliged to have abortions, and should not deny the majority from having reasonable access to the procedure.”

Finally, in the 2022 book *The Abortion Act 1967*, authors Sheldon et al. report that Steel was also pressured to include the clause by MP Norman St John-Stevas, whose chief loyalties were to the Catholic Church and Conservatism.^78^

## Exporting “Conscientious Objection” to Abortion Around the World

The Abortion Act was passed under a free vote (188 to 94) on October 27, 1967 and came into effect on April 27, 1968. In the last 58 years, the law has been amended several times, most notably a 1990 amendment to reduce the time limit for abortion from 28 to 24 weeks gestation.^79^ However, the “conscience clause” has not seen a single amendment since the initial adoption of the act.^80^

At least eleven jurisdictions incorporated the United Kingdom’s “conscientious objection” clause with only minor changes into their abortion laws. These include Barbados, Gibraltar (in 2019), Guernsey, Guyana, Jersey, Northern Ireland (in 2020), Seychelles, Singapore, South Australia, Tristan de Cunha, and Zambia. A few other jurisdictions loosely adopted the UK’s clause, including Isle of Man and Tasmania, Australia.

Whether the wording was similar or not, the idea caught on. Today, almost one hundred countries around the world have enacted laws or policies allowing belief-based care denial, according to a global map of belief-based care denial of abortion by REDAAS^81^ (Safe Abortion Access Network of Argentina, a network of health and legal professionals that provides abortion support) and a summary report of its findings.^82^

The REDAAS map shows that ten countries including the US allow “unlimited” belief-based care denial, eighty-seven countries put limits on the practice (but four of those allow institutional objection), and three countries explicitly disallow care denials based on personal beliefs.

Most measures that attempt to limit the negative impact of care denials do so by requiring objectors to make referrals, impart accurate information, and provide treatment in emergencies. However, these measures are rarely monitored or enforced and are widely disregarded.^83^ Meanwhile, the impact on patients is generally ignored and has been little studied.^84^

Ethiopia, Sweden, and Finland^85^ are the three countries that do not permit health care workers to deny care to patients based on their personal beliefs or “conscience.” Such refusals are explicitly disallowed through laws, policy, or court precedent, with apparent positive impacts. Potential objectors can find work in other fields, and disallowing care denials improves access to reproductive health services by reducing barriers and delays.

## Legal Cases Related to “Conscientious Objection”

In the United Kingdom, only two legal cases related to belief-based care denials have arisen since the passage of the 1967 *Abortion Act.* In 1988, a secretary objected to typing out letters referring patients to an abortion clinic.^86^ In 2014, two Catholic midwives objected to supervising other staff who were providing abortions.^87^ All plaintiffs lost their case on the basis that they were not directly involved in the abortion procedure.

Such cases are emblematic of the push by antiabortion campaigners to expand the ability to object beyond doctors and nurses and beyond actual abortion provision. In the wake of the midwives case, a UK bill was introduced and debated in 2018 that would allow doctors, nurses, midwives, and pharmacists to refuse to not only take part in abortions but also any activity “required to prepare for, support or perform termination of pregnancy.” The bill expired with the proroguing of the 2017–2019 session of the UK Parliament.^88^

At least three cases in which health care professionals tried to expand their ability to deny care based on their beliefs have been pursued in other countries. In 2015 in Sweden, where midwives routinely provide abortions as part of their duty of care, two antiabortion midwives separately sued their local health authority and county, respectively, because they were denied employment due to their refusal to provide abortions for “conscience” reasons. Both lost,^89^ with the European Court of Human Rights ruling in one of the cases that any infringement of the complainant’s freedom of religion did not violate the *European Convention on Human Rights*, because:

“Sweden … has a positive obligation to organise its health system … to ensure that the effective exercise of freedom of conscience of health professionals in the professional context does not prevent the provision of such services. The requirement that all midwives should be able to perform all duties inherent to the vacant posts was not disproportionate or unjustified.”^90^

In Canada, Christian medical groups and several antiabortion doctors sued the College of Physicians and Surgeons of Ontario in 2015 over its requirement that objectors provide an “effective referral” to a physician who can provide the service. They lost,^91^ with the Ontario Court of Appeal unanimously ruling that patients’ rights to equitable access to medical services outweighs a physician’s freedom to deny care on religious grounds.

In Uruguay in 2015, a number of doctors challenged Decree 375, which regulated the scope of the country’s liberal abortion law and placed limits on “conscientious objection” to guard against abuse. The doctors argued that the decree unduly restricted their right to freedom of thought, and the court agreed, annulling several of the limits around belief-based care denial. In particular, the Court voided the requirement that prohibited physicians from forming any value judgment on a patient’s decision. Reproductive rights advocates warned that the decision meant that any further gains made in the realm of sexual and reproductive rights would be thwarted.^92^

Globally, over a dozen patients or their families have either filed human rights complaints or sued health services or governments for harms done to them by health care workers denying care due to personal beliefs.^93^ A few cases succeeded, but most did not or are still ongoing. Rarely have doctors been held accountable. For instance, when Savita Halappanavar died in 2012 in Ireland after being denied a legal, life-saving abortion, Savita’s husband and her parents obtained financial settlements from the hospital and the responsible doctor for her wrongful death;^94^ however, no health care workers were criminally charged for the medical negligence in Savita’s case, nor did any lose their license. In Michigan in 2010, Tamesha Means was denied life-saving treatment at a Catholic hospital when she began to miscarry but lost her lawsuit when the court declared that her claim of negligence would “impermissibly intrude upon ecclesiastical matters.”^95^

In a rare case of accountability, seven doctors in Italy were tried for manslaughter in October 2019 because Valentina Milluzzo died of sepsis in 2015 after miscarrying twins at 17 weeks of pregnancy. Her parents testified that doctors refused to give her a termination because they declared themselves to be “conscientious objectors,” and the fetuses still had heartbeats. A decision in the case was announced in October 2022. Three doctors were acquitted and four were sentenced to six months each for manslaughter but received suspended sentences. It is not known whether the convicted doctors also lost their medical licenses.^96^

## Belief-based Care Denial as Practiced Today

### Situation in the UK

In the United Kingdom today, about 70% of NHS-funded abortions occur in private clinics dedicated to abortion care, mostly run by the British Pregnancy Advisory Service (BPAS) and MSI Reproductive Choices.^97^ Since their clinics only hire staff who support abortion provision, the “CO” clause in the Abortion Act is redundant in the case of private clinics. Therefore, belief-based care denials are primarily limited to public hospitals in the UK, as well as GP private practices.

The National Health Service^98^ has terms of service that require objectors to refer appropriately, as does the Royal College of Obstetricians and Gynaecologists.^99^ However, a 1999 article^100^ noted that a substantial minority of trainees in obstetrics-gynecology opt out of abortion training. The UK government changed requirements in 1994 to say that abortion duties cannot be included as part of a job advertisement, and applicants cannot be asked about their beliefs.^101^ In 2003, the Department of Health clarified that this guidance was not intended to cover “career posts exclusively for termination of pregnancy.”^102^

### Flouting of “CO” regulations by objectors

Despite requirements that objecting health care professionals must refer for abortion, significant numbers refuse to do so, whether in the UK or elsewhere.^103^ This reality is consistently overlooked when countries or medical bodies enact laws or policies requiring referral, which rarely include monitoring or enforcement mechanisms. Yet, objections to referral requirements were a subject of debate even before abortion was legalized in the UK. The BMJ published the Catholic view on abortion referrals in December 1966:^104^

“A practitioner who cannot in conscience entertain the request to procure abortion must not be required to name specific practitioners or clinics known to practise the operation, for thereby he becomes an accessory before the fact in what he considers to be murder.”

Regulatory authorities generally do not scrutinize care deniers, assuming they will obey directives such as referring appropriately and taking action in emergencies. Available evidence paints a different picture. Approximately eighty stories about women who suffered serious harm or injustice after being denied legal abortion, including death in several cases, were collected by the authors from media and NGO reports.^105^ These stories are the tip of an iceberg, as only a few cases ever become public. Most of the stories revealed that objectors tended to violate laws or policies meant to limit the denial of care and some may have committed malpractice. That is, belief-based care denials are usually accompanied by one or more of the following additional behaviors:

- Refusing to refer
- Failing to provide necessary information
- Lying to patients or providing misinformation
- Judging or criticizing patients
- Violating patients’ privacy
- Not listening to patients or dismissing their concerns
- Delaying patients; making them wait for treatment or tests
- Not attending to patients in hospital
- Not providing pain relief
- Failing to follow standard medical protocols
- Waiting till the patient is near death before acting

In the overall literature, a number of reports and articles describe injustices caused by belief-based care denials,^106^ with some of these publications authored by pro-choice researchers who support the practice but call for better regulation to mitigate the harms.^107^ However, no country has shown that it can successfully allow the practice of belief-based care denial while ensuring quality access to abortion care.

A single paper by Chavkin et al.^108^ has been frequently cited by supporters of “conscientious objection” to claim that some countries — specifically, Norway, England, and Portugal — can accommodate objectors while still assuring the provision of abortion services. However, a published response in 2017^109^ (by two of us) showed that the selection of these countries biased the conclusion. Norway, and other countries with low religiosity like Estonia,^110^ have small numbers of objectors, making belief-based care regulations largely redundant. In Portugal, there were indications that many hospitals were not following the regulations at that time, and in fact, a 2024 report revealed that belief-based care denial in Portugal is rampant, involving not just doctors but a range of healthcare workers refusing to have any association with abortion. Even though the law permits only individuals to be objectors and mandates public health establishments to provide abortion care, many health units had declared themselves to be an objector as a whole, and one third of hospitals were not doing abortions, along with every one of the 55 health centres approached by researchers.^111^

In England, private clinics provide most abortions, a workaround that bypasses objectors — at least for patients attending clinics. Ireland also found a workaround by operating a central registry where patients can call for a referral to an abortion provider, allowing them to bypass antiabortion doctors.^112^ This means that when the regulations governing “CO” play a minimal role or are sidelined, we cannot conclude they are working well. However, a January 2025 study revealed serious issues with belief-based care denial in the hospitals of England and Wales.^113^ Some of the study findings: The legal protection of “conscientious objection” enabled stigmatization of abortion including a lack of support for providers. The ability to deny care was used beyond its legal scope, with abortion care denied across entire services or hospitals. Belief-based care denial was employed selectively depending on the patient’s reason for abortion, the pregnancy’s gestational length, or if there was a fetal anomaly.

### Studies of patient experiences with care denials

Virtually no studies have been done anywhere in the world on what happens to patients who encounter an objector when they need an abortion.^114^ We found just three.

A 2021 study interviewed 121 women in Poland to examine their experiences as contraception purchasers at pharmacies.^115^ Nine percent of participants who tried to purchase contraceptives faced a refusal based on a pharmacist’s conscience clause, and 88 percent of participants said that pharmacists should not be entitled to refuse to sell contraceptives.

A 2023 study in the UK interviewed five service users who had been denied abortion care, using a narrative approach.^116^ The service users had made appointments with doctors who did not always disclose their objection to abortion, or did not provide enough information to access abortion, or treated them judgmentally, or provided medically incorrect information. Although all service users were later able to obtain abortion care, and one service user described a sensitive and respectful denial of care, four were left with negative emotions, including feeling scared, angry, and hopeless when they were not referred or were mistreated.^117^

Finally, a new study to be published in 2025 (co-authored by one of us) interviewed 30 participants who had been denied contraception or abortion in Canada. Participants experienced negative feelings of anger, fear, disappointment, or frustration due to the refusal; and consistently expressed opposition to policies that allow providers to refuse reproductive health services based on their beliefs.^118^

## Unprincipled Basis for “Conscientious Objection” in Healthcare Paved the Way for Harms

The overview of the history and aftermath of the “conscientious objection” clause in the 1967 Abortion Act reveals a disturbing connection between the origins and motivation behind the clause, and the harms that followed.

Members of the UK Parliament and the medical profession were aiming to preserve doctors’ authority over patients, protect objecting doctors from liability, and appease religious antiabortion beliefs. These factors point to an unprincipled basis for the introduction of “conscientious objection” into healthcare. Even though the 1967 Abortion Act legalized abortion and was a major advance at that time for the rights and health of women, the deep stigma of abortion and its prior illegal status meant that lawmakers centered the interests of physicians and the Catholic Church with the introduction of the “conscience clause.” This ultimately came at the expense of patients’ rights and health. The practice of denying health care for “conscience” reasons spread around the world and resulted in ongoing patient harms across many countries, including too-frequent grievous harms like permanent injury and death.

The “conscience clause” also represented a repudiation of some basic ethical directives in medical practice, specifically patient autonomy, shared decision-making, and fiduciary duty to patients — whose interests should be paramount. Further, the term “conscientious objection” — borrowed from the military but misapplied to healthcare — provided a moral façade for the practice of denying care to vulnerable patients based on a physician’s personal beliefs. This helped mask the practice as a “right” to protect doctors, even while it disregarded patients’ rights, health, and dignity.

### Continuum of harms caused by belief-based care denial

Regulations that limit belief-based care denials require objectors to provide emergency care, but some doctors will risk a pregnant person’s death rather than perform an abortion. In Poland, where abortion is still legal to save a pregnant woman’s health or life, doctors have let several patients die, rather than treat their pregnancy complications.^119^ The same happened in Ireland with Savita Halappanavar and in Italy with Valentina Milluzzo.^120^ A documentary film called “Abandoned” told the stories of these two women and others in Europe who were seriously harmed by belief-based care denial.^121^

In any case, the legal requirement to provide a service in a life-threatening situation does not work, for the simple reason that it is impossible to predict with certainty whether a medical case is truly life-endangering or when it will become so — until the patient actually dies. Differing medical opinions on the risk of death means that some will advise a “wait and see” approach until it is too late. These voices may be guided not by medical knowledge and skills, but by personal beliefs, particularly in countries hostile to abortion rights.^122^

The extent of harm caused varies but is often much worse than a short delay.^123^ Any denial of care inevitably creates some degree of harm to patients, ranging from inconvenience, humiliation, psychological stress, additional costs, delays in care, unwanted pregnancy, increased medical risks, and death.

Even where the harm appears to be minimal — for example, the care denier provides a referral and the patient receives prompt services — refusal of care can still be harmful because it can demean and shame them. It sends a stigmatizing message about the care they need, undermining their dignity and autonomy.

Therefore, refusing to provide legal and necessary care to patients amounts to a violation of their right to health and life, and their moral autonomy. The decades-long acceptance of “conscientious objection” and its prioritization of physicians’ interests has gone largely unquestioned until the 21^st^ century, demonstrating that the health care profession has for too long been blinded to the harms visited upon patients. Past and ongoing gender discrimination in healthcare, including pregnancy care, lends support to this observation.^124^

### Denial of abortion care as gender discrimination

Advocates for abortion rights argue that access to safe abortion has become a fundamental human right, and an ethical and necessary service that has saved countless lives^125^ and furthered their social, political, and economic equality.^126^

No other area of health care allows treatment denial based on a patient’s gender, race, religion, disability, or medical condition (except more recently, medical assistance in dying^127^). But social conservatism means that women are often expected to fulfil a motherhood role, and may face ignorance, disapproval, or hostility when requesting abortion. Belief-based care denial then becomes a paternalistic initiative to compel women to give birth. More seriously, it represents a repudiation of women’s autonomy and civil rights because it is not possible for women to achieve equality without access to abortion and contraception.^128^

Objections to providing abortions are based on a denial of women’s rights and harms of criminalization. The provision of safe, legal abortion is a vital public interest that negates any grounds for belief-based care denial.

### Role of abortion stigma and anti-abortion politics

Belief-based care denial is inextricably linked to abortion stigma and political action against abortion rights.^129^ From the start, the Catholic Church, conservative politicians, and antiabortion activists have sought to ensure that healthcare professionals and Catholic hospitals could deny abortion provision under the guise of “conscience,”^130^ even though that meant violating the personal consciences of pro-choice physicians working at Catholic hospitals.

The United States in particular serves as a cautionary tale. In recent years, “religious liberty” has become weaponized against women and the 2SLGBTQIA+ community, by sanctioning discrimination against them not only through the denial of abortion care or gender-affirming care, but also by refusals to dispense contraception, provide insurance coverage for birth control, offer supportive services for same-sex marriages, or bake a wedding cake or host a reception for a same-sex marriage.^131^ Further, religious and antiabortion organizations in the US and elsewhere have continually sought to expand the reach of health care denials and to immunize objecting health care workers from any consequences.^132^ In effect, laws and policies allowing belief-based care denial serve as an escape clause for anyone to boycott laws legalizing abortion (or prohibiting discrimination), and to disregard their professional obligations to patients.

### Misplaced assumptions behind the regulation of care denial

Limiting the exercise of belief-based care denial rests on the misconception that objecting health care personnel will accept and make the required compromises, including referring for abortion or providing accurate information on the procedure. For example, recommendations from the American College of Obstetricians and Gynecologists specify that objectors to reproductive care must fulfill their primary duty to the patient and keep the patient’s well-being paramount, impart accurate and unbiased information, disclose scientifically accurate information, refer patients in a timely manner to other providers, and provide the requested care in an emergency.^133^

However, such expectations rely on trusting practitioners to set aside deeply held personal beliefs that have already been deemed strong enough to invoke a “conscientious objection”, making any compromise far less likely. In fact, objectors often see no moral difference between doing an act and being complicit in it by referring or allowing it. As once stated by an antiabortion writer:^134^

“From the perspective of a doctor with a conscientious objection to abortion, referral to another practitioner is like saying, ‘I can’t rob the bank for you myself. But I know someone down the road who can.’ In other words, referral involves becoming complicit in the abortion. It is therefore something that health care practitioners with an objection to abortion rightly refuse to do.”

Because objectors often view a referral as nearly equivalent to doing the procedure themselves, limiting their “right” to object is contradictory and unacceptable from their point of view. They will frequently not follow limitations, facilitated by the lack of monitoring and the inability of patients to enforce their rights. For example, rather than impart accurate information based on science, antiabortion doctors may share misinformation and engage in moral judgments, dissuasion, delay and sometimes abuse.^135^ Many care deniers even follow a “conscience absolutism” doctrine, under which “the professional is not obligated directly or indirectly to participate in [a service] provision or facilitate patient access to it.”^136^ That means no information and no referral that might in any way lead the patient to the requested service.

We should also question whether religious beliefs should be allowed to interfere with the provision of necessary health care, given that best practices depend on scientific evidence and professional medical ethics. Once health care workers are allowed to make personal or “faith-based” decisions instead of evidence-based ones, it becomes impossible to regulate the practice of belief-based care denial or stop its expansion, since one cannot objectively challenge the sincerity or veracity of someone’s religious beliefs.^137^

Some scholars argue that allowing objectors to deny care is necessary to prevent their “moral distress” or protect their “moral integrity.”^138^ However, objectors have freely chosen their discipline along with its obligations to provide patient care, and their refusals cause moral distress to patients and harm *their* moral integrity.^139^

Others say the responsibility should shift to health systems and hospitals, which can ensure abortion provision while protecting individual objectors.^140^ This overlooks the ethical and logistical problems with belief-based care denial and cements them into place on a systems level. Why should the public system be expected to support health care workers who refuse to do their jobs?

Allowing care denials also overlooks the burden on non-objecting providers, who in countries like Italy may find themselves doing most of the abortions, forgoing some of their own desired duties or working overtime, and enduring stigma and harassment from colleagues.^141^ Why are care deniers allowed to opt out of part of their jobs and hand off their responsibilities to colleagues, while maintaining their full pay and status and facing little or no accountability?

### Care denial cannot be an exercise in “balancing” rights

A common argument in favor of belief-based care denial is the alleged need to find a “balance” between the rights of patients and the rights of health care professionals, thereby framing it as an issue of competing rights. This argument has a false premise — that a patient’s need for basic medical care is morally equivalent to protecting a health care provider’s personal beliefs. This notion trivializes the health of patients, particularly women and gender-diverse individuals needing abortion care. There is no “balance” when an authority figure is allowed to impose their beliefs on a powerless person who needs the services that only the person in power can provide. The patient is the one who pays the price and bears the burden of care denial in reproductive health care, not the health care provider.

Patients not only have a right to conscience, but also a right to life and health, liberty, equal protection, privacy, dignity, and other basic rights. These may all be denied in addition when a doctor vetoes their health care. A doctor’s right to freedom of conscience cannot outweigh or be balanced against this long list of fundamental human rights for patients.

## What Is the Proper Role of Conscience in Health Care?

Global human rights agreements recognize the right to conscience as a basic individual right, but as we have argued, the term “conscientious objection” in healthcare is inaccurate and misapplied and therefore should not be equated with the universal right of conscience. Indeed, international human rights agreements do not recognize “conscientious objection” in health care as a right and the United Nations, World Health Organization, Amnesty International, and Human Rights Watch have recognized its harms and called for limits on its exercise.^142^

Certainly, conscience does play a role in health care. When doctors join the profession, they agree to assume professional obligations to patients and follow medical codes of ethics. Doctors are bound by laws on negligence and by “fiduciary duty” — a legal or ethical relationship of confidence or trust between two or more parties. Patients cannot typically go elsewhere to obtain services because of the control that doctors have over provision of medical services. Therefore, when doctors cite their conscience as a reason to deny health care to a patient, they are disavowing their public responsibilities and violating their professional ethics — creating in effect a conflict of interest.^143^

Belief-based care denial must also be distinguished from legitimate acts of conscience in medicine that stem directly from healthcare workers’ obligations to their patients and to their professional ethics. Three key examples are: (1) “Conscientious commitment” — a term coined by Bernard Dickens^144^ that we define as the provision of necessary or beneficial health care to patients in need despite stigma, unjust laws, or oppressive systems; (2) The refusal to provide harmful or morally questionable “treatments” without genuine patient consent, including torture, executions, infant circumcision, or other non-beneficial care; and (3) The refusal of treatment based on the principle of “beneficence” or “non-maleficence” to ensure the patient is helped or at least not harmed,^145^ for example, if a patient requests a risky experimental treatment or a patient with mental health issues wants an unnecessary procedure such as an amputation.

Since the above actions are done in line with widely accepted medical ethics that respect patient needs or interests, and not because of subjective personal or religious beliefs, they do not fit the definition of belief-based care denial. Indeed, the latter should be considered morally asymmetrical to these true acts of conscience.^146^

When medical professionals personally oppose abortion or contraception, the only appropriate time for them to truly exercise their conscience in that regard is when they choose a discipline as a medical student. Objectors should simply not enter the fields of obstetrics and gynecology or family medicine. If they do, they are in effect forfeiting their conscience rights once they take an oath to put patients first.

## Conclusion

A survey of the origin of so-called “conscientious objection” in health care shows that it was fundamentally rooted in opposition to the self-determination of women who request abortion, having begun with the United Kingdom’s 1967 Abortion Act. The term was adopted despite the fact that denial of health care based on personal or religious beliefs has little in common with conscientious objection in the military.

The “conscience” clause in the Act was an artifact of abortion criminalization. Politicians and medical groups wanted to protect doctors who refuse to do abortions from liability, but their motivations were rooted in moral opposition to abortion, doctor authority and protectionism, and Catholic religious beliefs against abortion. While the UK’s law did give women legal access to safe abortion for the first time, priests at a Catholic seminary in Scotland played an outsized role in placing a limitation on the right to abortion from the outset. The “conscience” clause provided a precedent for medical practitioners to boycott a democratically decided law because of personal beliefs, and to be exempt from any liability. It also allowed doctors to uphold historic patriarchal authority over patients. Around the world, this has led to reduced access to abortion and violations of human rights, particularly in countries with a strong social stigma against abortion and a large number of objectors.^147^

Jonathan Montgomery stated in 2015 that “… ‘conscientious objection’ as set out in section 4 of the 1967 Abortion Act needs to be understood as an act of heresy, a departure from the orthodox professional identity….” He concluded: “… in a twenty-first century context, once the historical contingencies of the Abortion Act’s conscience clauses are recognised, the case for a specific exemption is dramatically reduced. It is no longer necessary for such a clause to be in place to secure collective medical support for abortion services.”^148^

A report from a 2017 international meeting on the topic, titled “Unconscionable: When Providers Deny Abortion Care” concluded that:^149^

“…health care policies should not allow for the refusal to provide services based on conscience claims. Where policy-makers are revising abortion laws or policies, they should not make references to conscience claims. Enshrining into law the notion that providers’ personal beliefs can determine the provision of health care opens up the door to abuses and legitimizes conscience claims.”

Dozens of scholars and researchers^150^ have presented evidence and arguments against belief-based care denial in any kind of health care. International human rights agreements do not explicitly recognize it as a right, and the United Nations and World Health Organization have acknowledged the harms it can cause.^151^

In the face of ubiquitous care denials, the self-managed abortion revolution featuring abortion pills has become a powerful new way to circumvent objectors. Groups such as Women on Web and Women Help Women allow people to purchase mifepristone and misoprostol over the Internet and safely navigate their own abortions. As Women Help Women states on its website:^152^ “Key to reproductive freedom and justice is putting abortion pills directly into the hands of those that need them.” Medication abortion has an excellent efficacy and safety record,^153^ including for its use in self-managed abortions.^154^ Despite this, many countries where abortion is legal impose severe restrictions on medication abortion,^155^ showing again that most governments do not want women to decide for themselves whether to terminate an unwanted pregnancy — the same reason that UK medical groups in 1967 supported the “conscientious objection” clause in the Abortion Act. Self-managed abortion therefore allows patients to bypass many barriers, including obligatory doctor visits as well as belief-based care denial.

However, doctors and hospitals will always be needed to provide abortion in clinical settings, so legislators and the medical profession must take action. As the instigator of so-called “conscientious objection” in health care, the United Kingdom should set an example for the rest of the world by amending Section 4 of the 1967 Abortion Act to disallow the denial of care based on personal beliefs, then urge other countries to follow suit.

When policymakers understand the dishonorable origin of “conscientious objection” in healthcare, the injustices that followed and multiplied across the world, and the inherent contradictions between medical ethics and belief-based care denial, they should be motivated to create change. We suggest shifting the narrative to one that recognizes belief-based care denial as a harmful and discriminatory tactic that should be discouraged. The practice can be phased out gradually using disincentives and other measures^156^ to encourage objectors to choose other fields, with the goal of eventually eliminating belief-based care denials. The experiences of Sweden and Finland^157^ prove this is not only possible but is the best way to safeguard the health and lives of women and others seeking abortion care.
